# TGFBR1*6A as a modifier of breast cancer risk and progression: advances and future prospects

**DOI:** 10.1038/s41523-022-00446-6

**Published:** 2022-07-19

**Authors:** Kojo Agyemang, Allan M. Johansen, Grayson W. Barker, Michael J. Pennison, Kimberly Sheffield, Hugo Jimenez, Carl Blackman, Sambad Sharma, Patrick A. Fordjour, Ravi Singh, Katherine L. Cook, Hui-Kuan Lin, Wei Zhang, Hui-Wen Lo, Kounosuke Watabe, Peiqing Sun, Carl D. Langefeld, Boris Pasche

**Affiliations:** 1 Department of Cancer Biology, Wake Forest School of Medicine, Winston-Salem, NC 27157-1082 USA; 2 Department of Thoracic Medicine, School of Medicine, University of Adelaide, Adelaide, SA 5000 Australia; 3 Comprehensive Cancer Center, Wake Forest School of Medicine, Winston Salem, NC 27157-1082 USA; 4 Department of Surgery, Wake Forest School of Medicine, Winston-Salem, NC 27157-1082 USA; 5 Center for Cancer Genomics and Precision Oncology, Wake Forest School of Medicine, Winston Salem, NC 27157-1082 USA; 6 Department of Biostatistics and Data Science, Wake Forest School of Medicine, Winston-Salem, NC 27157-1082 USA; 7 Center for Precision Medicine, Wake Forest School of Medicine, Winston-Salem, NC 27157-1082 USA

**Keywords:** Breast cancer, Oncogenesis

## Abstract

There is growing evidence that germline mutations in certain genes influence cancer susceptibility, tumor evolution, as well as clinical outcomes. Identification of a disease-causing genetic variant enables testing and diagnosis of at-risk individuals. For breast cancer, several genes such as *BRCA1, BRCA2, PALB2, ATM*, and *CHEK2* act as high- to moderate-penetrance cancer susceptibility genes. Genotyping of these genes informs genetic risk assessment and counseling, as well as treatment and management decisions in the case of high-penetrance genes. TGFBR1*6A (rs11466445) is a common variant of the TGF-β receptor type I (*TGFBR1*) that has a global minor allelic frequency (MAF) of 0.051 according to the 1000 Genomes Project Consortium. It is emerging as a high frequency, low penetrance tumor susceptibility allele associated with increased cancer risk among several cancer types. The TGFBR1*6A allele has been associated with increased breast cancer risk in women, OR 1.15 (95% CI 1.01–1.31). Functionally, TGFBR1*6A promotes breast cancer cell proliferation, migration, and invasion through the regulation of the ERK pathway and Rho-GTP activation. This review discusses current findings on the genetic, functional, and mechanistic associations between TGFBR1*6A and breast cancer risk and proposes future directions as it relates to genetic association studies and mechanisms of action for tumor growth, metastasis, and immune suppression.

## Heritable breast cancer genes

Heritable predisposition genes are important risk factors for breast cancer susceptibility, accounting for 5.03% of all breast cancer cases^1–4^. Pathogenic variants of high-risk predisposition genes such as *BRCA1* and *BCRA2* are the most widely known and are used in genetic testing and counseling to predict breast cancer risk and clinical outcomes^4,5^. However, these variants are uncommon (<1.3% allelic frequency), and account for less than 1.5% of all breast cancer cases^4,6^. Like other complex genetic traits such as diabetes, obesity, and autoimmune diseases, recent advances in genome-wide association studies reveal that the vast majority of hereditary breast cancer cases are genetically multifactorial^7–10^, involving numerous other polymorphisms of varying penetrance acting as tumor modifier genes^11–13^. Thus, several lower penetrance risk variants are now included in breast cancer susceptibility gene screening panels for testing and counseling. Inclusion of these polymorphisms into screening panels results in a 40 to 50% increase in breast cancer risk detection among women, and a 5 to 15% increase in detection among *BRCA1/2*-negative females^6,11,12,14^.

The Transforming Growth Factor-Beta (TGF-β) signaling pathway plays a critical role during cancer development and progression^15–17^. Variants of the TGF-β pathway genes, particularly TGFBR1*6A, have been studied in a large number of females, and have been associated with low penetrance risk for breast cancer^18^ (Table 1). This review details our current clinical and pre-clinical knowledge on TGFBR1*6A as a high frequency, low penetrance tumor susceptibility allele, and provides further rationale to assess its role as a modifier gene for breast cancer predisposition and tumor progression.

**Table 1:** Case-control studies showing TGFBR1*6A genotypic and allelic distribution and frequency.

Study	Country of study participants	Self-reported race/ethnicity	Genotype distribution and frequency (%)						Allelic frequency	
			Cases			Controls			Cases	Controls
			9/9A	9A/6A	6A/6A	9A/9A	9A/6A	6A/6A	TGFBR1*6A	*TGFBR1*
Pasche et al. 1999 (a)	US	Mixed	128 (84.2)	24 (15.8)	0 (0)	654 (89.3)	78 (10.7)	0 (0)	0.079	0.053
Pasche et al. 1999 (b)	Northern Italy	Caucasian	39 (81.3)	8 (16.7)	1 (2.1)	38 (76)	12 (24)	0 (0)	0.104	0.120
Baxter et al. 2002	United Kingdom	Caucasian	268 (75.5)	83 (23.4)	4 (1.1)	207 (83.5)	39 (15.7)	2 (0.8)	0.128	0.087
Reiss, 2004	US	Mixed	87 (88.8)	11 (11.2)	0 (0)	77 (84.6)	14 (15.4)	0 (0)	0.056	0.077
Caldes, 2004	Spain	Caucasian	214 (79)	56 (20.7)	1 (0.4)	250 (85.6)	42 (14.4)	0 (0)	0.107	0.072
Offit, 2004	US	NS	391 (84.6)	67 (14.5)	4 (0.9)	291 (88.2)	38 (11.5)	1 (0.3)	0.081	0.061
Northwestern, 2004	US	NS	74 (86.1)	12 (13.9)	0	105 (85.4)	17 (13.8)	1 (0.8)	0.070	0.077
Jin et al. 2004 (a)	Finland	Caucasian	177 (80.1)	38 (17.2)	6 (2.7)	171 (73.1)	60 (25.6)	3 (1.3)	0.113	0.141
Jin et al. 2004 (b)	Poland	Caucasian	140 (82.4)	28 (16.5)	2 (1.2)	176 (87.1)	26 (12.9)	0 (0)	0.094	0.064
Kaklamani et al. 2005	US	Mixed	515 (84.3)	92 (15.1)	4 (0.7)	612 (88.7)	77 (11.2)	1 (0.1)	0.082	0.057
Chen et al. 2006	US	Mixed	92 (80)	23 (20)	0 (0)	111 (85.4)	18 (13.8)	1 (0.8)	0.100	0.077
Feigelson et al. 2006^#^	US	Mixed	387 (80.5)	94 (19.5)^a^	NS	384 (74)	100 (26)^a^	NS	0.098	0.130
Cox et al. 2007	US	NS	968 (81.6)	207 (17.4)	12 (1)	1352 (80.8)	302 (18.1)	19 (1.1)	0.097	0.102
Song et al. 2007	Sweden	Caucasian	598 (78.4)	152 (19.9)	13 (1.7)	682 (80)	160 (18.8)	10 (1.2)	0.117	0.106
Jakubowska et al. 2009	Poland	Caucasian	282 (88.7)	33 (10.4)	3 (0.9)	252 (86.9)	38 (13.1)	0 (0)	0.061	0.066
Colleran et al. 2009	Ireland	Caucasian	796 (82.9)	154 (16)	10 (1)	785 (81.9)	160 (16.7)	13 (1.4)	0.091	0.097
Joshi 2011 (a)	India	Asian	163 (97.6)	4 (2.4)	0 (0)	213 (95.9)	9 (4.1)	0 (0)	0.012	0.020
Joshi 2011 (b)	India	Asian	33 (78.6)	8 (19)	1 (2.4)	148 (87.6)	19 (11.2)	2 (1.2)	0.119	0.068
Kamali et al. 2015^#^	Iran	Middle Eastern	251 (89.6)	25 (8.9)	4 (1.4)	241 (86.1)	27 (9.6)	12 (4.3)	0.059	0.091

Case-control studies from 1999 to date have included a total of 14,837 participants (6787 cases /8050 controls); of which 13,312 (6026 cases/7286 controls) were in Hardy–Weinberg (HW) equilibrium. The data shows genotype distribution and allelic frequency (%) in the order TGFBR1*9A/9A > TGFBR1*9A/6A > TGFBR1*6A/6A.

*NS* not stated.

^#^Study population not in Hardy–Weinberg equilibrium.

## TGFBR1*6A and breast cancer

### Identification and characterization

**Fig. 1:**
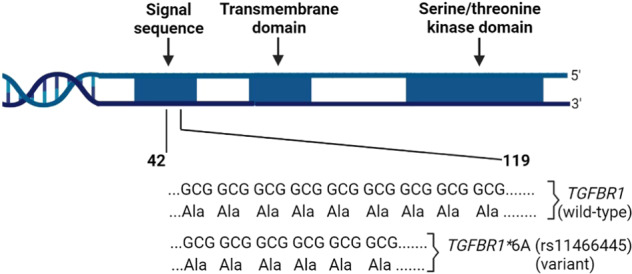
*TGFBR1* and TGFBR1*6A gene and protein sequences. Sequence analyses reveal nine (9) GCG/alanine repeats within nucleotides 42–119 of the *TGFBR1* signal sequence. TGFBR1*6A variant has six (6) GCG/alanine repeats in its signal sequence (Pasche, Luo et al. 1998, Pasche, Kolachana et al. 1999).

The TGF-β pathway functions as a tumor suppressor during cancer development but enhances tumor growth, immune evasion, and metastasis in advanced cancers^15–17^. During TGF-β signaling, the TGF-β receptor 1 (TGFBR1) plays a critical role of binding and activating downstream receptor-regulated (R-) SMADs (SMAD2 and SMAD3), co-SMADs (SMAD4)^19,20^, and non-SMAD (MAPK-ERK, RAS, AKT, JNK, and RHOA)^17,21^ intermediary proteins. In 1998, Pasche et al. identified TGFBR1*6A (rs11466445) as a polymorphic variant of *TGFBR1* with an in-frame deletion of three GCG codons encoding alanine within exon 1 of the human *TGFBR1* signal peptide sequence^22,23^ (Fig. 1). Signal peptides are responsible for intracellular transport, targeting of nascent proteins (secretory and membrane proteins) to the endoplasmic reticulum, and integration of newly translated proteins into their respective compartments. N-terminal signal peptides are usually cleaved off and degraded after insertion and targeting of the nascent protein to the endoplasmic reticulum^24^. Investigations into TGFBR1*6A signal peptide cleavage showed that the 3-alanine deletion from the 9-alanine repeat within the hydrophobic core of the signal peptide does not affect the posttranslational cleavage of a signal peptide. Studies showed that TGFBR1*6A signal peptide is cleaved between Ala30 and Leu31, whereas the wild-type TGFBR1 is cleaved between Ala33 and Leu34. Importantly, the mature forms of the TGFBR1*6A and TGFBR1 receptors are identical and the TGF-β ligand binds to each receptor with the same affinity^23,25^. Also, the differences in cleavage sites do not influence TGFBR1*6A protein targeting and translocation functions^25^ nor its sensitivity, and half-life^23^. Despite TGFBR1*6A and TGFBR1 physicochemical similarities, TGFBR1*6A is intriguingly associated with breast cancer risk^26,27^, and promotion of cell growth, migration, and invasion^25,28^. Only the released signal peptide separates TGFBR1*6A from TGFBR1, which strongly suggests that TGFBR1*6A signal peptide contributes to oncogenesis.

## Genetic association with breast cancer risk

### Case-control studies

#### Allelic frequency

TGFBR1*6A (rs11466445) is a common variant of the TGF-β receptor type I (*TGFBR1*) with a study-wide minor allelic frequency (MAF) of 0.051 in the 1000 Genomes Project Consortium, ranging from 0.0079 among East Asians to 0.0975 among Europeans^29–31^. Nineteen case-control studies that included 14,837 participants (6787 cases/8,050 controls) have investigated the association of TGFBR1*6A with breast cancer risk (Table 1). Seventeen of these studies, which included 13,312 individuals (6026 cases/7286 controls), show the TGFBR1*6A genotype frequencies were in Hardy–Weinberg equilibrium (HWE). HWE is a useful genotyping quality control metric that relates allele frequencies to genotype frequencies with an expected assumption that genotype frequencies will remain constant in a randomly mating population. The afore-mentioned TGFBR1*6A case/control studies consisted predominantly of Caucasian individuals of European ancestry (>80%), but also included modest numbers of individuals from other self-reported races/ethnicities (e.g., African American, Hispanic, Asian, and Middle Eastern). Early studies examined a mixed sample of Caucasians, African Americans, Hispanics, and Asians from the US, and Northern Italy, and found an allelic frequency of 7.9% in breast cancer patients compared to 5.3% in healthy controls^23^. Several other studies from the US consisting of >80% Caucasians and <20% non-Caucasians reported similar TGFBR1*6A allelic frequency of 5.6–10.0% among breast cancer patients^26,32–35^. The TGFBR1*6A allelic frequency also ranged from 6.1 to 12.8% among breast cancer patients from northern and southwestern Europe, including samples from the UK^36^, Spain^32^, Sweden^37^, Finland^38^, Poland^38,39^, and Ireland^40^ (Table 1). In all, Asian and Middle Eastern women, particularly those from Western India had the lowest TGFBR1*6A allelic frequency, 1.2–11.9% among cases^41^. In all, TGFBR1*6A genotype frequency is in the order 9A/9A > 9A/6A > 6A/6A in all populations studied to date (Table 1)^23,38,41^.

#### Risk association

**Fig. 2:**
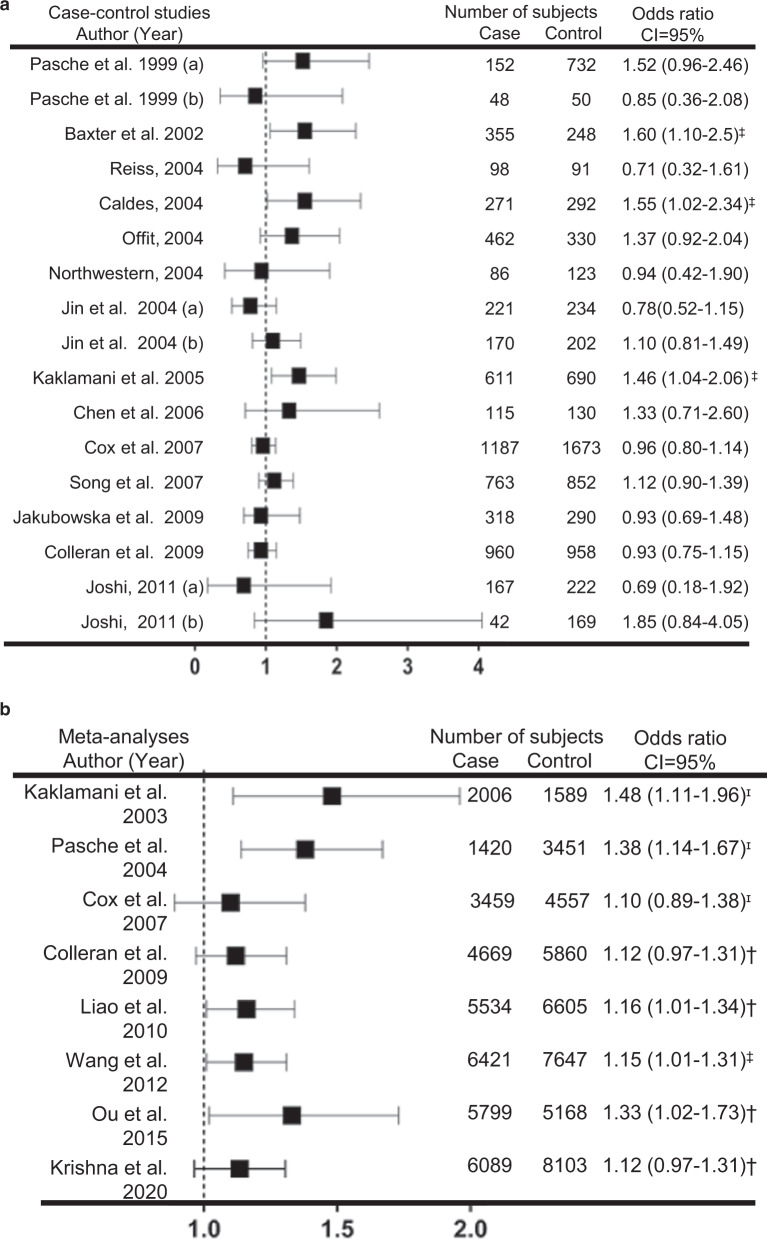
Studies investigating TGFBR1*6A association with breast cancer risk. Forest plot showing the number of subjects and odds ratios of **a** Case-control studies, and **b** Meta-analyses associating TGFBR1*6A to breast cancer risk. Plot **a** includes only case/control studies that are in Hardy–Weinberg equilibrium. CI confidence interval, ɪ dominant association (*p* ≤ 0.01), ‡ additive association (*p* ≤ 0.05), † allelic association (*p* ≤ 0.05).

Published case-control studies examining the association of TGFBR1*6A with breast cancer risk among individuals from different geographical locations and ethnicities show both significant and non-significant risk associations^26,41,42^. It remains unclear if the differences in TGFBR1*6A risk association correlate with geographical location, ethnicity, age, tumor stage, and other confounding factors such as other polymorphism frequencies, lifestyle, and environment. In 2002, Baxter et al. reported the first association between TGFBR1*6A and breast cancer risk (OR 1.6, 95% CI 1.1–2.5)^36^. Cases and controls were residents of Southampton, UK. Controls were healthy females. Breast cancer cases were selected based on age at onset under 40 years, family history of breast cancer irrespective of age at onset, or bilateral breast cancer irrespective of family history or age at onset. The study noted that TGFBR1*6A allelic frequency among Caucasians from the UK does not differ by age of onset (<40 years), bilateral breast cancer, family history, and germline mutations of *BRCA1* and *BRCA2*^36^. In 2004, Caldes investigated breast cancer patients of Caucasian descent only from Madrid, Spain. The study found a significant association between TGFBR1*6A and breast cancer risk (OR 1.55, 95% CI 1.02–2.34)^32^. In the Kaklamani 2005^26^ study that included >80% Caucasians from New York, NY, TGFBR1*6A association with breast cancer risk was significant under both dominant (OR 1.50, 95% CI 1.07-2.11) and additive (OR 1.46, 95% CI 1.04-2.06) models. Breast cancer risk for women aged >50 years was higher (OR 2.20, 95% CI 1.25–3.87), than for women aged ≤50 years (OR 1.18, 95% CI 0.75–1.84). However, further subgroup analyses show no association between TGFBR1*6A and ER/PR status or cancer stage at diagnosis. In this study, both breast cancer patients and healthy controls were matched for age, gender, and location^26^. Other studies that included participants of similarly mixed ethnicities but from other states in the United States did not confirm an association between TGFBR1*6A and breast cancer risk^32,33,35^ (Fig. 2a). This indicates that TGFBR1*6A association with breast cancer risk may have a more modest effect size than in the original reports (regression toward the mean) and these studies were not powered to account for the effects of other confounding/modifying factors apart from ethnicity and geographical location that modify the magnitude of the risk attributable to the TGFBR1*6A polymorphism.

Intriguingly, other studies have investigated participants of similar Caucasian backgrounds from other European countries (Sweden, Ireland, Finland, and Poland) and found no significant associations (Fig. 2a)^37–40^. In a case-control study among Swedish Caucasians^37^, the study further enriched its population sampling for genetic susceptibility by recruiting patients with family history of bilateral breast cancer cases. Overall, TGFBR1*6A carriership was not associated with breast cancer risk (OR 1.12, 95% CI 0.90–1.39). However, subgroup analyses showed that TGFBR1*6A was associated with increased risk among low-risk familial breast cancer patients with one first- or second-degree breast cancer relative (OR 1.3, 95% CI 1.0–1.9). In that study, TGFBR1*6A also correlated with higher tumor grade (OR 2.27 (95% CI 1.01–5.11) but had no association with tumor stage or ER/PR status^37^. For women of Asian and Middle Eastern descent, TGFBR1*6A association with breast cancer risk was neither significant nor protective^41,42^. Among Asians from India, Joshi 2011^41^ found no association among Western Indians (OR 0.69, 95% CI 0.18–1.92) and those from the Parsi community (OR 1.85, 95% CI 0.84–4.05)^41^. Although these are important studies, the sample sizes are modest, and the lower allelic frequency in this population^43^ results in very low a priori statistical power to detect an association of the size previously reported. To date, TGFBR1*6A appears to be associated with decreased breast cancer risk only in Middle Eastern women, particularly in Iran (OR 0.62, 95% CI 0.39–0.98)^42^. However, it is worth noting that the genotype frequencies in this sample of 280 cases and 280 controls show strong deviations from Hardy–Weinberg equilibrium expectations (*X*^2^ *p* value = 2.81E-12), an important measure of genotyping quality. Specifically, there were an excess of both TGFBR1*6A and *TGFBR1* homozygotes, with a corresponding deficit of heterozygotes, when compared to HWE expectations.

In summary, it appears that TGFBR1*6A is a common breast cancer susceptibility allele in diverse ethnic backgrounds and its influence may vary by ancestry, geographical location, and other risk modifying factors. Large case/control studies, with important related phenotypes, paralleling the magnitude of other complex genetic traits, and meta-analyses that incorporate strict genotype quality control assessment will be necessary to clarify its population-specific subgroup risk associations.

## Meta-analyses of TGFBR1*6A association with breast cancer risk

To derive a more precise estimate of TGFBR1*6A association with breast cancer risk, several meta-analyses have investigated its risk association in up to 14,837 participants (6787 cases/8050 controls) from 19 case-control studies. In all, eight meta-analyses have studied the association of TGFBR1*6A with breast cancer risk (Fig. 2b). Five of the studies found evidence that supports the association between TGFBR1*6A and breast cancer risk. Early meta-analyses by ref. ^44^ and ref. ^32^ that analyzed up to 12 case-control studies (4871 subjects) found an association of TGFBR1*6A with breast cancer risk with odds ratios of 1.48 (95% CI 1.11–1.96) and 1.38 (95% CI 1.14–1.67), respectively. Subsequent studies examined ten case/control studies and did not find a significant risk association (OR 1.10, 95% CI 0.89–1.38), albeit in a concordant direction. The heterogeneity in the reported results may be partly due to meaningful differences in the genotyping methods employed across these studies for this GCG repeat polymorphism, and increased variation in estimates due to lower sample sizes included in the case/control studies^35^.

Three of the most recent meta-analyses that investigated up to 17 case/control studies (14,068 subjects) found an association between TGFBR1*6A and breast cancer risk. Liao et al. 2010 reported an odds ratio of 1.16 (95% CI 1.01–1.34) among 11,220 case/controls^45^. Wang et al. 2012 investigated 14,068 participants and showed a nearly identical OR of 1.15 (95% CI 1.01–1.31) ^27^. Additionally, Ou et al. 2015 found 14.8 and 9.6% TGFBR1*6A allelic distribution among 6275 cases and controls, respectively. There was a significant association of the TGFBR1*6A allele with breast cancer risk (OR 1.33 95% CI 1.02–1.73)^46^. The study also noted that the homozygous TGFBR1*6A/6A is not significantly associated with breast cancer risk. Colleran 2010^40^ and Krishna 2020^47^ found no significant associations between TGFBR1*6A and breast cancer risk (Fig. 2b). It is interesting to note that the latter two meta-analyses selectively excluded some data from the Pasche 2004, and other case/control studies in their analyses. For example, case/control studies by refs. ^32,39^, all of which are in Hardy–Weinberg equilibrium were selectively omitted from the ref. ^35^, ref. ^40^ and ref. ^47^ meta-analyses. Colleran et al., 2010 explained that some study populations from Jin et al., Reiss, and Offit had been reported in Kaklamani et al. 2005, and as such omitted them in their analyses. However, these study populations were different and there was no overlap with the population reported in Kaklamani et al. 2005 and prior reports. Additionally, Colleran et al., measured heterogeneity in the case/control studies used in their meta-analyses, and, noted that studies with sample size less than 1000 had the most extreme odds ratios, indicating odds ratio heterogeneity among different case/controls as another contributing factor^40^. Overall, most meta-analyses published to date show an association between TGFBR1*6A and breast cancer risk. Further studies are warranted to establish the risk association and its relatedness to different population sub-groups.

## TGFBR1*6A functional effects

### Promotion of cell proliferation, migration, and invasion

Physicochemical studies using R1B/L17 and HEK 293 cells revealed that the mature TGFBR1*6A and the wild-type TGFBR1 receptors have similar half-life, receptor turnover, and binding affinity to TGF-β ligand^23,25^. In MCF-7 breast cancer cells stably transfected with either *TGFBR1* or TGFBR1*6A, TGF-β/SMAD signaling was comparable^28^. However, the stably transfected TGFBR1*6A MCF-7 breast cancer cells exhibited enhanced cell growth, migration, and invasion^25,28^. Importantly, TGFBR1*6A switched TGF-β anti-proliferative effects into growth stimulatory effects in the MCF-7 breast cancer cells. The TGFBR1*6A-mediated switch to growth stimulation was independent of TGFBR1*6A kinase domain, indicating a mechanism likely due to TGFBR1*6A signal peptide^25^. A similar conclusion was also reached following the investigation of TGFBR1*6A induction of migration and invasion. There was a 1.2-fold and 1.7-fold increase in migration and invasion, respectively, in TGFBR1*6A MCF-7 cells when compared to *TGFBR1* transfected cells^28^. In the MCF-7 cells expressing low TGFBR1*6A levels, there were 1.3 and 1.9 times increase in migration and invasion respectively, when compared to the vector controls. Cells expressing higher and intermediate TGFBR1*6A levels showed 1.8 and 2.2 times increase in migration and invasion, respectively. The induction of migration and invasion observed in the TGFBR1*6A cells were not affected by TGF-β stimulation, suggesting an underlying migration mechanism that is TGF-β independent^28^. Similar observations of increased growth and invasion were reported in colorectal cancer cells that endogenously harbor TGFBR1*6A. Using SW48 (*TGFBR1*/*TGFBR1*) and DLD1 (TGFBR1/TGFBR1*6A) cells, TGF-β treatment resulted in growth inhibition in the *TGFBR1/TGFBR1* SW48 cells while it resulted in growth stimulation in the TGFBR1/TGFBR1*6A DLD1 cells^25^. Stable transfection of the colorectal cancer cells with TGFBR1*6A also exhibited enhanced proliferation when compared with the vector-transfected cells^48^, indicating that TGFBR1*6A has similar functional outcomes in breast and colorectal cancer.

## Mechanism of action

**Fig. 3:**
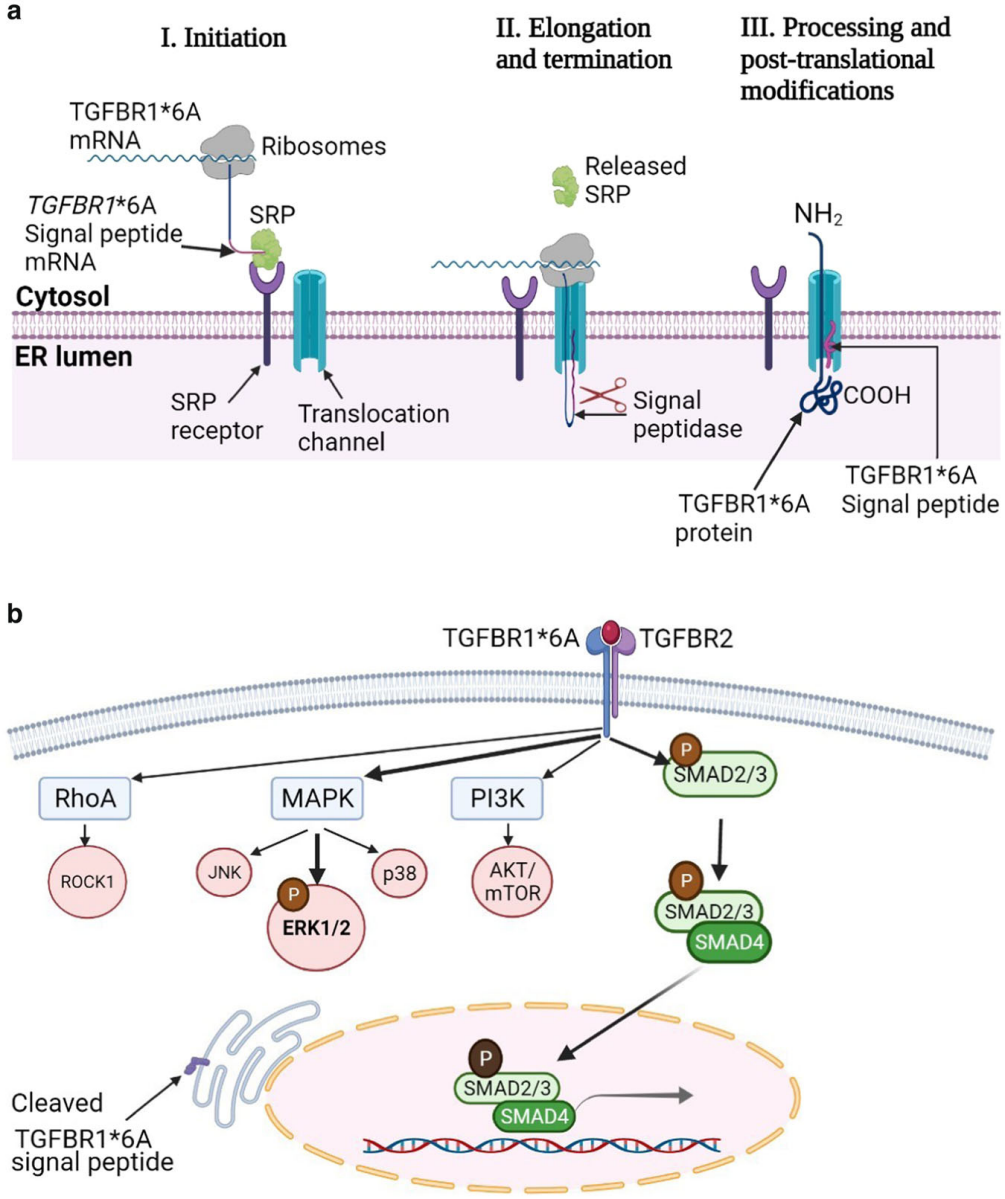
Schematic showing the role of TGFBR1*6A signal peptide. **a** TGFBR1*6A protein translation and processing, the TGFBR1*6A signal peptide is cleaved between Ala30 and Leu31, whereas the wild-type TGFBR1 is cleaved between Ala33 and Leu34. Both TGFBR1*6A and TGFBR1 wild-type exhibit similar binding affinity to TGFB ligand and stability (half-life). The TGFBR1*6A signal peptide also demonstrates similar protein targeting and translocation functions as the wild-type. **b** TGFBR1*6A intracellular signaling, TGFBR1*6A maintains intact TGF-β signaling to induce growth and migration in breast cancer cells. It shows similar TGF-β signaling as wild-type TGFBR1 but enhances phosphorylation of ERK1/2 to induce its tumor-promoting effects.

The functional and mechanistic understanding of tumor susceptibility alleles are important in establishing their clinical significance. TGFBR1*6A function in breast cancer cells is postulated to be mediated by its cleaved signal peptide, which is 3-alanine shorter than the wild-type TGFBR1 signal peptide (Figs. 1, 3a)^25^. Thus far, available evidence using SBE4-lux and 3TP-lux luciferase reporters suggest a mechanism that is not mediated by the mature TGFBR1*6A receptor. The SBE4-lux luciferase reporter assesses the binding of activated SMAD2/3/4 with SMAD binding elements in the nucleus as a measure of TGF-β/SMAD activation^28,49^. Likewise, 3TP-lux transcription reporter measures the binding of activated SMAD2/3/4 to three consecutive 12-*O*-tetradeca-noylphorbol-13-acetate (TPA) response elements (TREs) and a portion of the plasminogen inhibitor-1 (PAI-1) promoter region^28,50^. In a study that assessed the proliferation of MCF-7 cells expressing intermediate and high levels of TGFBR1*6A with kinase-inactivated domains, TGFBR1*6A enhanced cell proliferation in both clones similar to the kinase-activated TGFBR1*6A MCF-7 clones. Inactivation of the kinase domain did not influence TGFBR1*6A growth stimulation, indicating a cell proliferation enhancing mechanism that is not affected by the TGFBR1*6A kinase domain. Analyses of the TGF-β/SMAD signal transduction system using SBE4-lux and 3TP-lux transcription reporters, and phosphorylated-SMAD2 and SMAD3 levels showed comparable TGF-β/SMAD signaling levels for TGFBR1*6A and *TGFBR1* cells^25,28^. The absence of TGFBR1*6A influence on TGF-β signaling is consistent with the observed TGF-β independent effect on migration and invasion in MCF-7 cells. Investigations into TGF-β/non-SMAD pathways revealed that TGFBR1*6A increases ERK1/2 phosphorylation but shows no significant influence on p38 and JNK activation^28^. The upregulation of ERK1/2 phosphorylation is suggestive of MAP-kinase-mediated migration and invasion (Fig. 3b). A similar increase in MAP-kinase activation has also been correlated with increased invasion of TGFBR1*6A-transfected colorectal cancer cells. Zhou et al. showed that TGFBR1*6A expression enhances proliferation and invasion in stably transfected SW48 and DLD1 cells accompanied by increased ERK1/2 phosphorylation^48^. In contrast to breast cancer cells, the study noted an increase in p38 activation in the colorectal cancer cells. In all, the identified functional and mechanistic responses in colorectal cancer cells appears to be TGF-β-dependent^48^, indicating cancer-type differences in response mechanisms.

Further investigations into the signaling network of TGFBR1*6A-induced MCF-7 cell migration and invasion using Affymetrix GeneChip Human Genome U133 Plus 2.0 Array identified *ARHGAP5* and *FN1* as the key differentially expressed genes. The levels of *ARHGAP5* and *FN1* were downregulated in TGFBR1*6A MCF-7 cells when assessed with RT-qPCR and western blot assays^28^. *ARHGAP5* encodes the Rho GTPase activating protein 5 that negatively regulates the Rho-family of small GTPases. The Rho-family of small GTPases are small guanosine triphosphatases (GTPases) of the rat sarcoma (Ras) superfamily that function as molecular switches for cytoskeletal remodeling during cell division, cell-cell adhesion, cell contractility, migration, and invasion. *ARHGAP5* (P190B) and *ARHGAP35* (P190A) are the main regulators of the Ras homolog (Rho) family of actin-based regulators and are implicated in cellular adhesion, migration, and invasion^51,52^. *ARHGAP5* silencing inhibits migration and invasion of AGS and MGC-803 gastric adenocarcinoma cells^53^. In colorectal cancer, however, ARHGAP5 is markedly overexpressed in the liver of metastatic tissues compared to matched primary tumor tissues^54^. *ARHGAP5* suppression leads to decreased wound healing, migration, and invasion in DLD1 and SW480 colorectal cancer cells^54^. Similarly, amplification of *ARHGAP5* on the 14q12 chromosomal locus promotes the spreading and migration of Huh-7 hepatocellular carcinoma cells^55^. *FN1* encodes fibronectin, a soluble glycoprotein that binds to the cell surface and extracellular matrix. It is involved in cell-cell adhesion, cell motility, and maintenance of cell shape. Positive stromal fibronectin expression is significantly associated with low metastatic spread among patients with invasive breast carcinoma^56^. On the other hand, relatively low expression of stromal fibronectin correlates with lymph node metastasis, TNM stage, recurrence, and mortality^57^. Studies have also shown that tamoxifen-induced TGF-β expression regulates fibronectin levels in a feedback loop. TGF-β treatment reduced the levels of tamoxifen-induced *FN1* in MCF-7 cells^58^. Overall, the unbiased gene expression analysis revealed that TGFBR1*6A alters the expression of known pro- and anti-metastatic effectors, which may contribute to its effect on breast cancer progression.

## Perspectives and future directions

### Genetic association with breast cancer risk and progression

The case/control studies and meta-analyses assessing TGFBR1*6A association with breast cancer risk suggest an association between TGFBR1*6A and risk for breast cancer, albeit with demonstrable heterogeneity in odds ratios^26,27,32,41^. They also suggest that TGFBR1*6A-associated breast cancer risk may depend on ancestry, geographical location, and other confounding factors. In genetic association studies, there are primarily four possible explanations that account for a statistical association between a genetic polymorphism (e.g., TGFBR1*6A) and risk for a disease (e.g., breast cancer). They include (i) the allele directly affects the actual disease phenotype/expression, (ii) the allele is in linkage disequilibrium (correlated) with the true disease allelic locus, (iii) there is a spurious association due to population stratification or other confounders^59,60^, and (iv) type II error. To assert a direct allelic effect on disease phenotype/expression, the study design must control for confounding factors such as population stratification that could lead to false-positive/spurious associations. In general, ancestral population substructure is the main confounding concern contributing to spurious genetic associations^61,62^. Other sources include age, other mutations, and disease-specific contributory factors^13,59,63–65^. By design, population-based case/control studies such as those used in most of the TGFBR1*6A case/control studies are susceptible to spurious risk associations due to their random or convenient selection of cases and controls from participants that may not belong to the same ancestral population^66–69^. On the other hand, family-based case/control studies can help control for confounding population stratification because of shared family pedigrees;^59,70^ albeit these designs can be more difficult to recruit. Thus, in a bid to conclusively establish the risk association between TGFBR1*6A and breast cancer, future case/control studies should consider multicenter family-based population studies.

For multicenter population-based case/control studies that draw subjects from different self-reported races/ethnicities, careful consideration should be given to methods such as (i) structured association tests, (ii) principal components analyses (PCA), and (iii) multidimensional scaling (MDS) from GWAS ancestry to control genetic admixture and confounding effects^63,71–73^. Additionally, the effect sizes reported for nearly all complex genetic traits require large sample sizes for robust power and precise estimation. Thus, future studies should be designed with the range of magnitude of the effect, and allele frequencies observed in the published studies, and recruit cases and controls accordingly to enable well-designed and well-powered subgroup analyses of the contribution of the homozygous and heterozygous TGFBR1*6A genotypes. This will allow assessment of TGFBR1*6A risk association by age, ethnicity, family history, tumor subtypes, tumor grade/histology, other mutation (*BRCA1/2*) status, response to cancer treatment, and survival/mortality outcomes. When compared to high (*BRCA1* and *BRCA2*: odds ratios ranging from 5.0 to 10.6) and moderate (*CHEK2* and *ATM*: odds ratios ranging from 2.1 to 2.5) penetrance gene variants^4,74,75^, TGFBR1*6A has been described as low penetrance (<odds ratios <2.0) tumor susceptibility allele^27,36^. Although low penetrance gene variants and SNPs are common, their genetic risk to complex diseases such as cancer are comparatively small; carriers of low penetrance breast cancer gene variants are estimated to have less than 20% lifetime risk of developing breast cancers^75^. As such, low penetrance gene variants are postulated to exert their oncogenic influence through additive or multiplicative covariance interactions with other residual gene variants, whose presence or absence can determine disease trait^76,77^. Thus, the coupling of the TGFBR1*6A family-based case/control studies with the participants’ GWAS data will allow an additional estimation of the genome-wide composite association of TGFBR1*6A with breast cancer risk, as it relates to other gene variants and gene loci that are in linkage disequilibrium, polygenic association or epistatic interaction. Altogether, the estimation of the genetic risk and polygenic risk score of TGFBR1*6A from a robust family-based study will inform clinical guidelines for early detection and adoption of preventive measures.

## TGFBR1*6A mechanism of action and influence on TGF-β biomarker and drug development

Over the last decade, efforts aimed at developing anti-TGF-β drugs has for the most part led to inconclusive pre-clinical and clinical results and serious adverse events. This is at least partly due to imprecise biomarkers and drug targets identified so far. For example, in a recent phase I study of LY3022859^78^ (an anti-TGFBR2 kinase inactivating monoclonal antibody) in patients with advanced solid tumors including breast carcinomas, there were associated infusion-related reactions such as cytokine release syndrome. Also, the TGF-β small molecule inhibitor galunisertib (LY2157299) and several others acting as neutralizing monoclonal antibodies^79,80^ for TGFB1 and TGFB2, and protein traps^81^ for TGFBR1 and TGFBR2^82^ invariably yielded promising but minimal clinical benefits. In all, a key weakness is a difficulty to identify precise biomarkers that would tailor TGF-β inhibitors to a specific patient population. Recent studies have proposed and developed a TGF-β gene response signature (TBRS) to assess TGF-β signaling response as a biomarker for cancer predisposition, clinical outcome, and therapeutic response. It utilizes up to 153 genes as probes, which include mainly the TGF-β superfamily ligands (*TGFB1, BMP2*), TGF-β receptors (*TGFBR1* and *TGFBR2*), transcription factors (*BACH1, TXNIP,* and *CREB1*), and TGF-β responsive genes (*ID1, HMOX1, MMP2,* and *ZEB1*)^83–85^. Padua, 2008^83^ and Wahdan-Alaswad 2016^85^, used the TGF-β gene response signature (TBRS) to classify breast tumors as TGF-β gene responsive (TBRS+) and TGF-β gene unresponsive (TBRS-) breast tumor subtypes and found a higher correlation between TBRS+, and ER-^83^ and TNBC^85^ breast tumor subtypes. Additionally, recent studies relate TGF-β signaling with several cross-talk pathways including microRNA synthesis, stromal fibrosis, and endoplasmic reticulum (ER) stress that regulate immune checkpoint inhibitors, stem cell formation, and metastasis^86–90^.

The question of whether TGFBR1*6A functions singularly or in combination with other pro-oncogenic pathways to signal modifications in oncogenic traits needs to be answered to provide clues for tailored drug and biomarker development for the affected TGFBR1*6A individuals^91–95^. First, it is imperative to firmly establish whether TGFBR1*6A signaling is a signal peptide or receptor-mediated in various breast cancer subtypes and cell lines. Subsequent interactions and networking with identified cross-talk pathways and mediators can be traced using recent investigational tools such as Whole Transcriptome RNA Sequencing (RNA-seq) and proteomic profiling techniques coupled with computational network analyses such as comparative gene ontology (GO) enrichment analyses, ingenuity pathway analyses (IPA), and protein–protein interaction (PPI) network analyses^96–98^. In all, the development of a unique TGF-β gene response signature (TBRS) for TGFBR1*6A as well as the elucidation of a druggable target from the TGFBR1*6A transcriptomic and proteomic studies will be valuable for the prognosis of at-risk individuals and clinical evaluation for precision medicine.

## Concluding remarks

Genetic association studies have identified TGFBR1*6A as a high frequency, low penetrance breast cancer susceptibility allele in breast cancer patients of varied ethnic backgrounds and geographical locations. Its oncogenic influence is attributed to the promotion of cell proliferation, migration, and invasion. Further investigations into the risk associated with individual TGFBR1*6A genotypes as well as their correlation with breast cancer subtypes, disease progression (tumor grade), metastasis, and survival are needed to clarify the population-specific susceptibilities of TGFBR1*6A carriers.

## Data Availability

Any additional dataset other than cited published data were available upon request to the corresponding author.
